# Efficacy and safety of niraparib in patients aged 65 years and older with advanced ovarian cancer: Results from the PRIMA/ENGOT-OV26/GOG-3012 trial

**DOI:** 10.1016/j.ygyno.2024.03.009

**Published:** 2024-06-03

**Authors:** Giorgio Valabrega, Bhavana Pothuri, Ana Oaknin, Whitney S. Graybill, Ana Beatriz Sánchez, Colleen McCormick, Jean-François Baurain, Anna V. Tinker, Hannelore Denys, Roisin E. O’Cearbhaill, Sakari Hietanen, Richard G. Moore, Anja Ør Knudsen, Thibault de La Motte Rouge, Florian Heitz, Tally Levy, Whitney York, Divya Gupta, Bradley J. Monk, Antonio González-Martín

**Affiliations:** a AO Ordine Mauriziano Torino and Department of Oncology, University of Torino, Torino, Italy; b GOG Foundation and Departments of Obstetrics/Gynecology and Medicine, Division of Gynecologic Oncology, Laura & Isaac Perlmutter Cancer Center, NYU Langone Health, New York, NY, USA; c Vall d'Hebron University Hospital and Vall d'Hebron Institute of Oncology (VHIO), Barcelona, Spain; d Medical University of South Carolina, Charleston, SC, USA; e Unit of Genetic Counseling in Cancer and Gynecologic Oncology, Hospital General Universitario de Elche, Elche, Spain; f Legacy Medical Group Gynecologic Oncology, Portland, OR, USA; g Université Catholique de Louvain and Cliniques Universitaires Saint-Luc, Brussels, Belgium; h BC Cancer Vancouver, University of British Columbia, Department of Medicine, Vancouver, BC, Canada; i Department of Medical Oncology, Ghent University Hospital, Ghent, Belgium; j Gynecologic Oncology Group (GOG) and Department of Medicine, Memorial Sloan Kettering Cancer Center, Weill Cornell Medical College, New York, NY, USA; k Department of Obstetrics and Gynecology, Turku University Hospital, Turku, Finland; l Division of Gynecologic Oncology, Wilmot Cancer Institute, Department of Obstetrics and Gynecology, University of Rochester, Rochester, NY, USA; m Odense University Hospital, Odense, Denmark; n Department of Oncology, Eugene Marquis Cancer Center, Rennes, France; o AGO Study Group; Department for Gynaecology and Gynaecologic Oncology, Kliniken Essen-Mitte, Essen, Germany; Department of Gynaecology, Charité–Universitätsmedizin Berlin and Humboldt-Universität zu Berlin, Berlin, Germany; and Berlin Institute of Health, Berlin, Germany; p Department of Obstetrics and Gynecology, Wolfson Medical Center, Tel Aviv Faculty School of Medicine, Tel Aviv University, Holon, Israel; q GSK, Philadelphia, PA, USA; r GSK, Waltham, MA, USA; s Divison of Gynecologic Oncology, HonorHealth Research Institute, University of Arizona, Creighton University, Phoenix, AZ, USA; t Medical Oncology Department, Cancer Center Clínica Universidad de Navarra, Madrid, Program in Solid Tumours, CIMA, Pamplona, and Grupo Español de Investigación en Cancer ginecológicO (GEICO), Madrid, Spain.

**Keywords:** Ovarian cancer, Niraparib, PARP inhibitor, Age, Maintenance

## Abstract

**Objective.:**

To evaluate the impact of age on the efficacy and safety of niraparib first-line maintenance therapy in patients with newly diagnosed advanced ovarian cancer with a complete/partial response to first-line platinum-based chemotherapy.

**Methods.:**

Post hoc analysis of the phase 3 PRIMA/ENGOT-OV26/GOG-3012 study (NCT02655016). Patients in the intent-to-treat population were categorized according to age at baseline (<65 years vs ≥65 years), and progression-free survival (PFS), safety, and health-related quality of life (HRQOL) were evaluated for each age subgroup (clinical cutoff date, May 17, 2019). Safety findings were also evaluated according to a fixed starting dose (FSD) or an individualized starting dose (ISD).

**Results.:**

Of 733 randomized patients, 289 (39.4%) were ≥65 years (190 niraparib, 99 placebo) at baseline. Median PFS (niraparib vs placebo) and hazard ratios (95% CI) were similar in patients aged <65 years (13.9 vs 8.2 months; HR, 0.61 [0.47–0.81]) and ≥65 years (13.7 vs 8.1 months; HR, 0.53 [0.39–0.74]). The incidences of any-grade and grade ≥3 treatment-emergent adverse events (TEAEs) were similar across age subgroups; in the niraparib arm, TEAEs leading to dose discontinuation occurred in 7.8% of patients <65 years and 18.4% of patients ≥65 years. ISD use lowered the incidence of grade ≥3 thrombocytopenia events in niraparib-treated patients compared with the FSD (<65 years: 42.8% vs 18.0%; ≥65 years 57.0% vs 26.1%). HRQOL was comparable across age subgroups.

**Conclusion.:**

Niraparib efficacy, safety, and HRQOL were generally comparable across age subgroups, although patients ≥65 years had a higher rate of discontinuations due to TEAEs. ISD use reduced grade ≥3 thrombocytopenia events regardless of age.

## Graphical Abstract

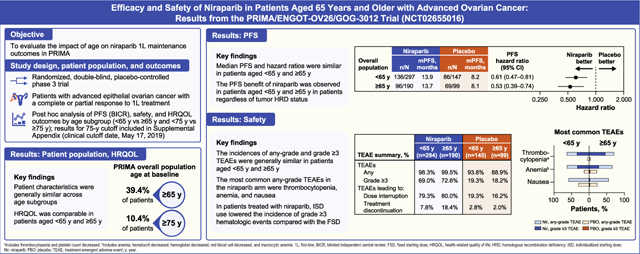

## 1. Introduction

Age is a well-established risk factor for ovarian cancer (OC), and incidence rates for OC increase with age worldwide [1,2]. In the United States, the median age for diagnosis of OC is 63 years, and approximately one-fifth of patients with OC are ≥75 years of age [3]. Increased age and factors associated with advanced age, including advanced International Federation of Gynecology and Obstetrics (FIGO) stage, comorbidity status, and poor tumor cytoreducibility, have all been shown to be independent prognostic factors for survival in patients with OC [4–6]. Although patients with germline *BRCA*-mutated (*BRCA*m) disease tend to be diagnosed at a younger age than patients with *BRCA* wild-type (*BRCA*wt) disease overall, this difference appears to be driven primarily by patients with *BRCA1* mutations [7,8].

Poor survival outcomes in older patients with OC are well documented, and studies indicate that these patients often receive suboptimal treatment compared with younger patients [9–11]. Although the reasons for potential suboptimal treatment in older patients are complex, concerns regarding tolerability are often foremost despite evidence supporting the use of both cytoreductive surgery and chemotherapy irrespective of age at diagnosis [9,10,12]. In a registry-based analysis of patients with advanced-stage epithelial OC, combination therapy use increased over time in patients aged 70–79 years and ≥80 years who received active treatment (2002–2004 vs 2008–2010) but remained lower than patients <70 years of age [13].

With the expansion of the OC treatment landscape to include maintenance treatment with poly(ADP-ribose) polymerase (PARP) inhibitors, it has become important to understand the tolerability and efficacy of these agents across age subgroups in both the primary and recurrent treatment settings. The PARP inhibitor niraparib was first evaluated in patients with platinum-sensitive recurrent OC in the ENGOT-OV16/NOVA (NOVA) trial [14]. In NOVA, niraparib maintenance therapy extended progression-free survival (PFS) compared with placebo [14], did not negatively impact health-related quality of life (HRQOL) [15], and had similar efficacy and safety in patients aged <70 years and patients aged ≥70 years [16]. Subsequently, niraparib was evaluated in the first-line setting in the PRIMA/ENGOT-OV26/GOG-3012 (PRIMA) trial. In PRIMA, maintenance treatment with the PARP inhibitor niraparib improved PFS compared with placebo in patients with newly diagnosed OC who responded to first-line platinum-based chemotherapy (PBC) regardless of biomarker status [17]. In this post hoc analysis of PRIMA, we evaluated the impact of age on the efficacy and safety of niraparib first-line maintenance therapy.

## 2. Methods

### 2.1. Study design

The study design and primary analysis results from PRIMA have been published previously [17]. Briefly, PRIMA is a double-blind, placebo-controlled phase 3 trial that evaluated niraparib in patients aged ≥18 years with newly diagnosed, advanced, high-grade serous or endometrioid ovarian, primary peritoneal, or fallopian tube cancer, with a complete or partial response to first-line PBC. All patients were required to provide tumor samples for homologous recombination (HR) testing (myChoice^®^ HRD test; Myriad Genetics, Inc., Salt Lake City, UT) and were eligible regardless of test results. Within 12 weeks of the first day of the last cycle of chemotherapy, patients were randomized 2:1 to receive either niraparib or placebo orally once daily (QD) in 28-day cycles until progressive disease or intolerable toxicity. At study start (July 2016), patients initially received a fixed starting dose (FSD) of 300 mg QD. Following a protocol amendment in November 2017, patients received an individualized starting dose (ISD) based on baseline body weight and baseline platelet count (200 mg QD for patients with body weight <77 kg or platelet count <150,000/μL; 300 mg QD for patients with body weight ≥77 kg and platelet count ≥150,000/μL). PFS assessed by blinded independent central review (BICR) was the primary efficacy endpoint. PRIMA was conducted in accordance with the tenets of the Declaration of Helsinki, Good Clinical Practices, and all local laws under the auspices of an independent data and safety monitoring committee; all patients gave informed written consent [17].

### 2.2. Outcomes

The primary objective of this post hoc analysis was to assess the efficacy, safety, and HRQOL in patients who received niraparib first-line maintenance treatment within each age subgroup. Patients were categorized by age at baseline, and 2 different age splits were examined based on a review of the clinical literature [16,18–22]: <65 vs ≥65 years and <75 vs ≥75 years. Duration of PFS assessed by BICR was a prespecified trial endpoint and was defined as time from randomization to earliest date of objective disease progression. Progression was assessed by computed tomography or magnetic resonance imaging every 12 weeks until treatment discontinuation according to Response Evaluation Criteria in Solid Tumors, version 1.1 (RECIST v1.1) [23]. Safety was assessed by age subgroup for treatment-emergent adverse events (TEAEs) reported in ≥20% of niraparib-treated patients for any age subgroup and by starting dose (FSD vs ISD) for medically important TEAEs of interest: thrombocytopenia, anemia, neutropenia, and hypertension. Adverse events were graded according to the National Cancer Institute Common Terminology Criteria for Adverse Events, version 4.03.

Patient HRQOL was a prespecified secondary endpoint and was evaluated using the European Organisation for the Research and Treatment of Cancer Quality of Life Questionnaire Core Questionnaire (EORTC-QLQ-C30) [24–27], the EORTC OC module (EORTC-QLQ-OV28) [26,28,29], the Functional Assessment of Cancer Therapy Ovarian Symptom Index (FOSI) [30,31], and the EQ-5D-5L [32,33]. Patient-reported outcome (PRO) assessments were performed at baseline (defined as the most recent measurement prior to the first administration of the study drug including day 1 of cycle 1), every 8 weeks (±7 days) for 56 weeks, and every 12 weeks (±7 days) thereafter while receiving study treatment. PRO assessments were also collected at the time of treatment discontinuation and at 4, 8, 12, and 24 weeks after the last dose of study treatment.

### 2.3. Statistical analysis

PFS was analyzed with a stratified log-rank test using stratification factors from randomization and summarized using Kaplan-Meier methodology. Hazard ratios with 95% CIs were estimated using a stratified Cox proportional hazards model, with stratification factors used in randomization (best response to first-line therapy [complete or partial response], receipt of neoadjuvant chemotherapy [yes or no], and tumor HR deficiency status [HR deficient (HRd) versus HR proficient (HRp) or HR not determined]). Multivariate Cox proportional hazards models were used to identify independent patient characteristics associated with PFS in patients <65 years and patients ≥65 years who received niraparib; this analysis was not performed in patients aged <75 years versus ≥75 years because of the small number of patients ≥75 years. Variables included in the initial model were Eastern Cooperative Oncology Group performance status (ECOG PS) scores (0 vs 1), FIGO disease stage (III vs IV), postoperative residual disease status (visible vs no visible residual disease), and all stratification factors used in randomization. A backward selection procedure was used, and only variables with a significance level <5% were selected for the final model.

For all PROs, least squares (LS) mean change from baseline was estimated using a mixed-effects model for repeated measures to adjust for data variability (detailed methods published previously, see Pothuri B et al. *Gynecol Oncol*, 2024;184:168–177. for additional details [34]). LS mean change from baseline (95% CI) over time data are reported for EORTC-QLQ-C30 (global health and overall QOL), EORTC-QLQ-OV28 (abdominal/gastrointestinal symptoms), FOSI, and EQ-5D-5L (visual analog scale [VAS]). Additional EORTC QLQ-C30 data for select functional scales (physical function) and symptom domains (fatigue, nausea and vomiting, pain, appetite loss, constipation, and diarrhea) are reported for patients <65 years and ≥65 years only; data are not reported for patients <75 years and ≥75 years because the small number of patients aged ≥75 years resulted in a level of variability that precluded meaningful interpretation of the results. This was a post hoc analysis, and the study was not powered to determine a treatment difference across age subgroups for evaluated endpoints. All analyses were conducted using data from the primary analysis data cut with a clinical cutoff date of May 17, 2019, and were performed using SAS^®^ 9.4 (Cary, NC).

## 3. Results

### 3.1. Patient population

The median age at baseline was 62 years (minimum, 32 years; Q1, 54 years; Q3, 69 years; maximum, 88 years). Of 733 randomized patients, 289 (39.4%) were aged ≥65 years and 76 (10.4%) were aged ≥75 years. Because of the limited number of patients aged ≥75 years (54 niraparib, 22 placebo), this analysis focused on patients aged <65 years (297 niraparib, 147 placebo) and patients aged ≥65 years (190 niraparib, 99 placebo). When available, results from the 75-year cutoff analysis are presented in the supplemental appendix.

The baseline demographic and clinical characteristics for the overall PRIMA population have been previously published [17]. Patient characteristics were generally similar between age subgroups for disease stage at baseline, best response to first-line PBC, postoperative residual disease, and tumor *BRCA2* mutation status. Compared with patients aged <65 years, a higher percentage of patients aged ≥65 years had ECOG performance status score of 1 (niraparib, 10.8% difference; placebo, 6.8% difference), neoadjuvant chemotherapy (niraparib, 8.1% difference; placebo, 11.4% difference); HRp tumors (niraparib, 13.0% difference; placebo, 11.5% difference; Table 1). Conversely, a higher percentage of patients aged <65 years had tumors with *BRCA1* mutations than patients aged ≥65 years (niraparib, 20.7% difference; placebo, 17.4% difference). Baseline characteristics for patients using the 75-year cutoff are shown in Table S1.

### 3.2. Efficacy

**Fig. 1.**
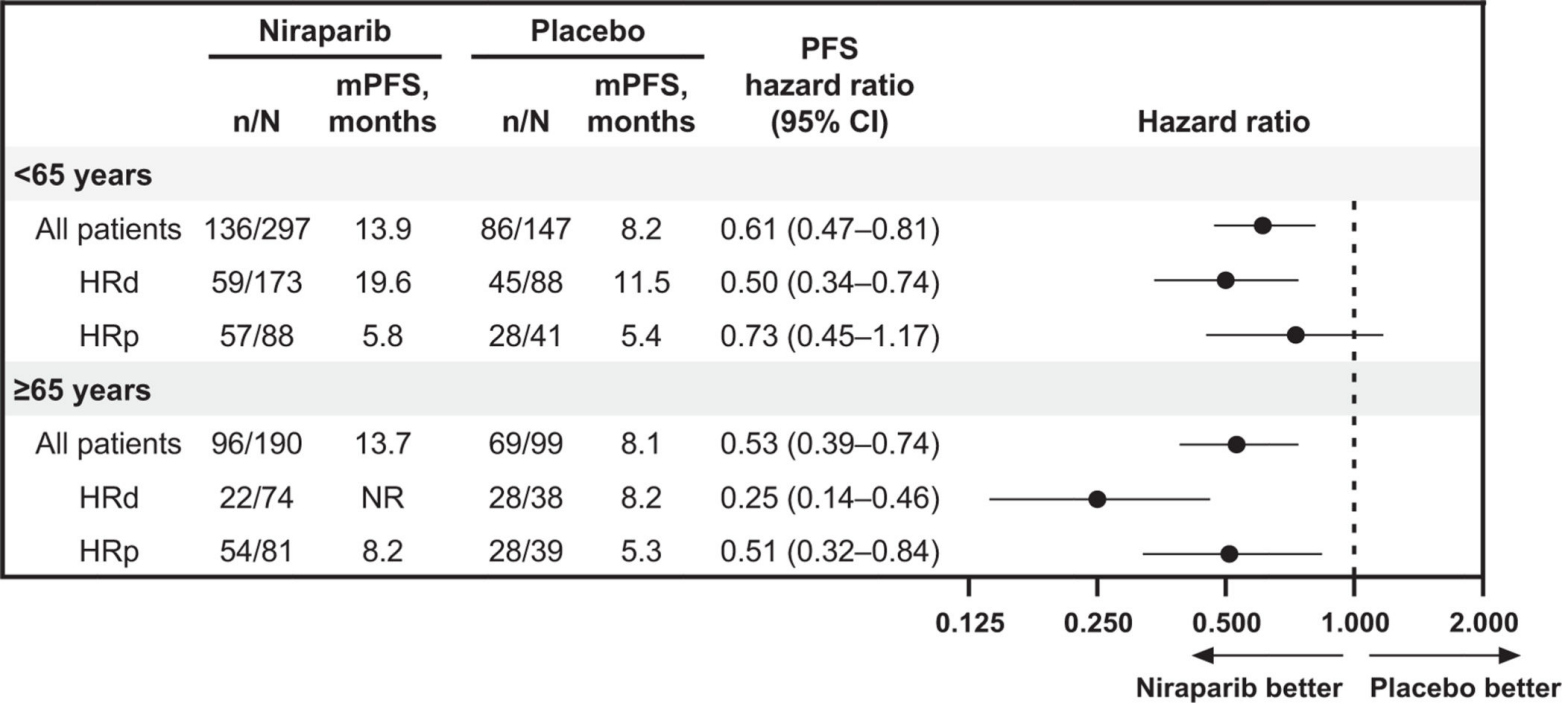
Efficacy (PFS) outcomes by age (<65 years and ≥65 years) and HRD status. Abbreviations: HRD = homologous recombination deficiency, HRd = homologous recombination deficient, HRp = homologous recombination proficient, m = median, NR = not reached, PFS = progression-free survival.

Niraparib efficacy was similar in patients aged <65 years and in patients aged ≥65 years, with longer median PFS (mPFS) in niraparib-treated patients than in placebo-treated patients. In patients aged <65 years, the mPFS was 13.9 months in the niraparib arm compared with 8.2 months in the placebo arm (hazard ratio, 0.61; 95% CI, 0.47–0.81); in patients aged ≥65 years, the mPFS was 13.7 months in the niraparib arm and 8.1 months in the placebo arm (hazard ratio, 0.53; 95% CI, 0.39–0.74; Fig. 1). PFS results also favored niraparib in patients aged <75 and ≥75 years in the overall population (Fig. S1). In patients with HRd tumors, the benefit of niraparib compared with placebo was maintained in both age subgroups; for patients <65 years, the hazard ratio was 0.50 (95% CI, 0.34–0.74), and for patients ≥65 years, the hazard ratio was 0.25 (95% CI, 0.14–0.46). In patients with HRp tumors, PFS hazard ratios were 0.73 (95% CI, 0.45–1.17) for patients aged <65 years and 0.51 (95% CI, 0.32–0.84) for patients aged ≥65 years (Fig. 1). In the multivariate analysis of PFS in patients who received niraparib, tumor HRd status remained in the model after backward selection and was significantly associated with improved PFS in both age subgroups (*P* < 0.0001; Table S2). In patients aged ≥65 years who received niraparib, best response to first-line treatment (complete vs partial response) was also retained in the model and was significantly associated with improved PFS (*P* = 0.0010).

### 3.3. Safety

The median duration of follow-up and the median treatment exposure were similar in the <65-year and ≥65-year age subgroups (Table 2). In each treatment arm, the incidences of any-grade and grade ≥3 TEAEs were similar in patients aged <65 years and ≥65 years (Table 2). In niraparib-treated patients, the incidence of serious TEAEs was 29.9% and 35.8% in the <65-year and ≥65-year subgroups, respectively. TEAEs leading to dose discontinuation in the niraparib arm occurred in 7.8% of patients aged <65 years and 18.4% of patients aged ≥65 years. In the 35 patients aged ≥65 years who discontinued niraparib because of a TEAE, 10 patients (28.6%) discontinued because of events that were grade 1 or 2 in severity (see Table S3 for additional details on TEAEs leading to niraparib discontinuation in patients aged ≥65 years). Results were similar when patients were assessed using the 75-year cutoff (Tables S3 and S4).

**Fig. 2.**
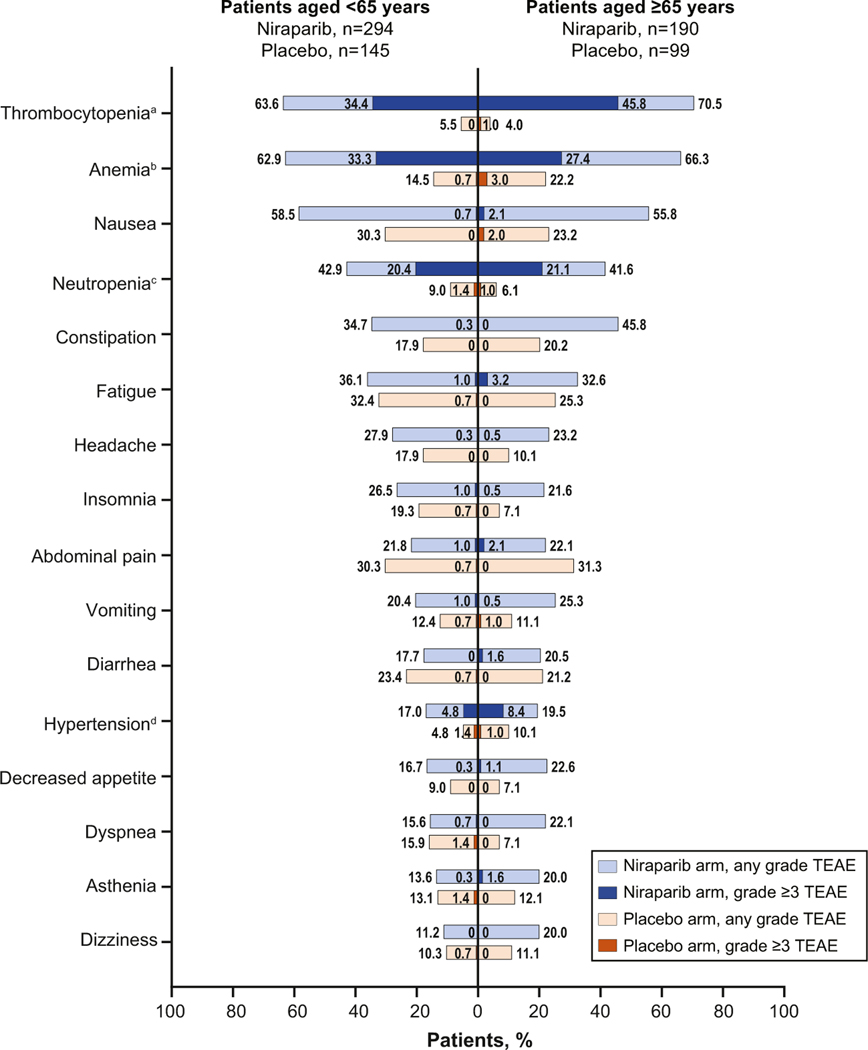
Most common TEAEs by treatment arm in patients aged <65 years and aged ≥65 years. TEAEs reported in ≥20% of niraparib-treated patients for any age subgroup (<65 years, ≥65 years, <75 years, ≥75 years). ^a^ Includes thrombocytopenia and platelet count decreased. ^b^ Includes anemia, hematocrit decreased, hemoglobin decreased, red blood cell decreased, and macrocytic anemia. ^c^ Includes neutropenia, neutrophil count decreased, neutropenic sepsis, and febrile neutropenia. ^d^ Includes hypertension and blood pressure increased. Abbreviations: TEAE = treatment-emergent adverse event.

In patients aged <65 years and ≥65 years, the most common any-grade TEAEs in the niraparib arm were thrombocytopenia, anemia, and nausea (Fig. 2). The most common grade ≥3 TEAEs with niraparib treatment were thrombocytopenia (<65 years, 34.4%; ≥65 years, 45.8%), anemia (<65 years, 33.3%; ≥65 years, 27.4%), and neutropenia (<65 years, 20.4%; ≥65 years, 21.1%). One patient in the niraparib arm, aged <65 years, developed myelodysplastic syndrome (MDS). In the <75-year and ≥75-year subgroups, the most common any-grade TEAEs in niraparib-treated patients were thrombocytopenia, anemia, and nausea; the most common grade ≥3 TEAEs were thrombocytopenia, anemia, and neutropenia (Fig. S2). In the placebo arm, which is generally considered reflective of the underlying comorbidities in the baseline population, the most common any-grade TEAEs in patients aged <65 years and ≥65 years were fatigue, abdominal pain, and nausea (Fig. 2). The most common any-grade TEAEs with placebo treatment were fatigue, abdominal pain, and nausea in patients aged <75 years and abdominal pain, nausea, and constipation in patients aged ≥75 years. No placebo-treated patients experienced events of MDS or acute myeloid leukemia (AML).

**Fig. 3.**
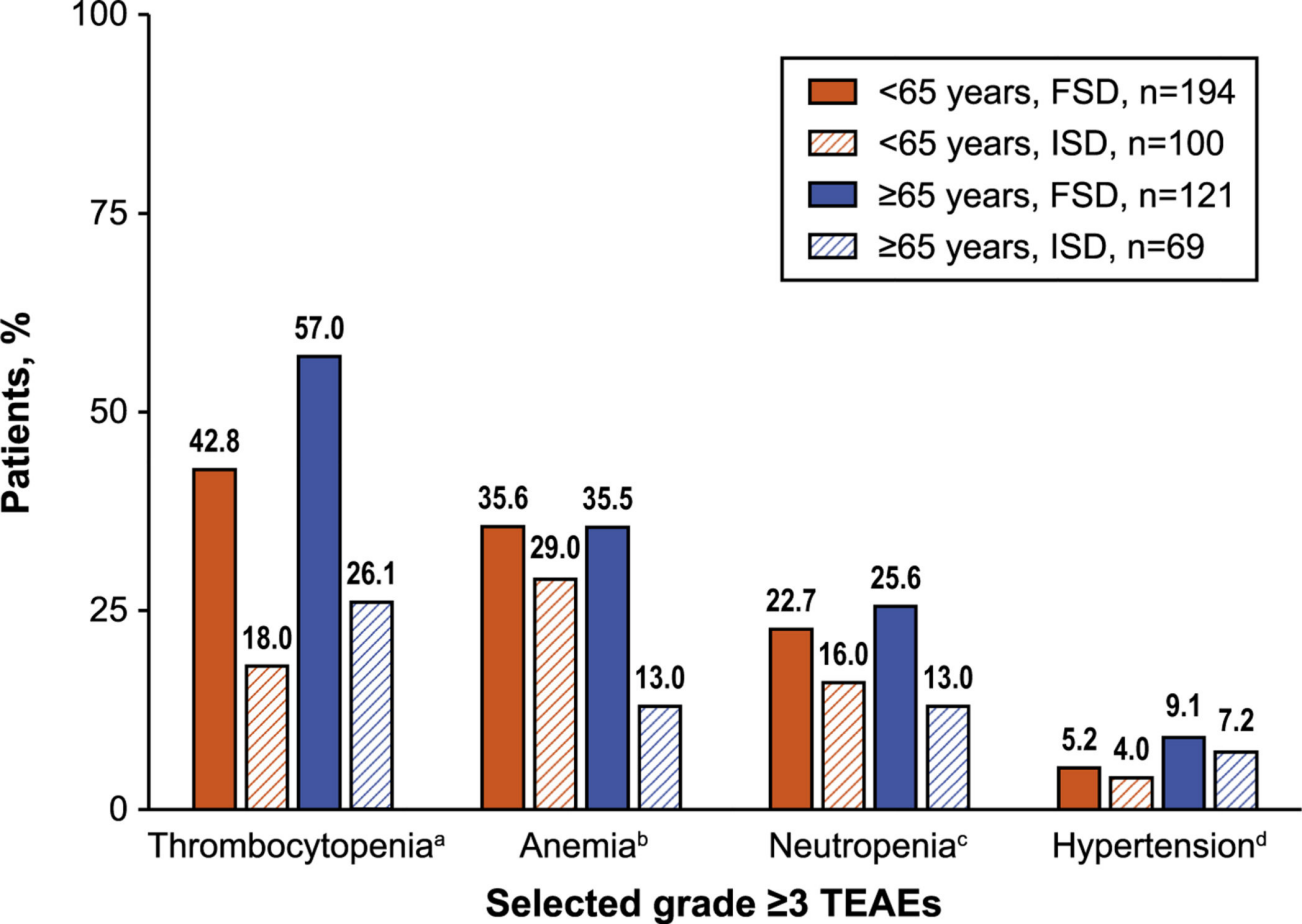
Selected grade ≥3 TEAEs in niraparib-treated patients by age (<65 years vs ≥65 years) and starting dose (FSD vs ISD). ^a^ Includes thrombocytopenia and platelet count decreased. ^b^ Includes anemia, hematocrit decreased, hemoglobin decreased, red blood cell decreased, and macrocytic anemia. ^c^ Includes neutropenia, neutrophil count decreased, neutropenic sepsis, and febrile neutropenia. ^d^ Includes hypertension and blood pressure increased. Abbreviations: FSD = fixed starting dose, ISD = individualized starting dose, TEAE = treatment-emergent adverse event.

To evaluate the effect of starting dose on safety, TEAEs in the niraparib arm were summarized according to whether patients received an FSD or an ISD based on baseline body weight and platelet count. Rates of grade ≥3 thrombocytopenia events were 42.8% with the FSD and 18.0% with the ISD in patients aged <65 years and 57.0% with the FSD and 26.1% with the ISD in patients aged ≥65 years (Fig. 3). Grade ≥3 anemia events occurred in 35.6% and 29.0% of patients aged <65 years who received the FSD and ISD, respectively; in patients aged ≥65 years, rates were 35.5% and 13.0% by starting dose. Similar trends in reduced rates of selected grade ≥3 TEAEs with ISD use were also observed in patients aged <75 years and ≥75 years (Fig. S3).

### 3.4. Patient-reported outcomes

**Fig. 4.**
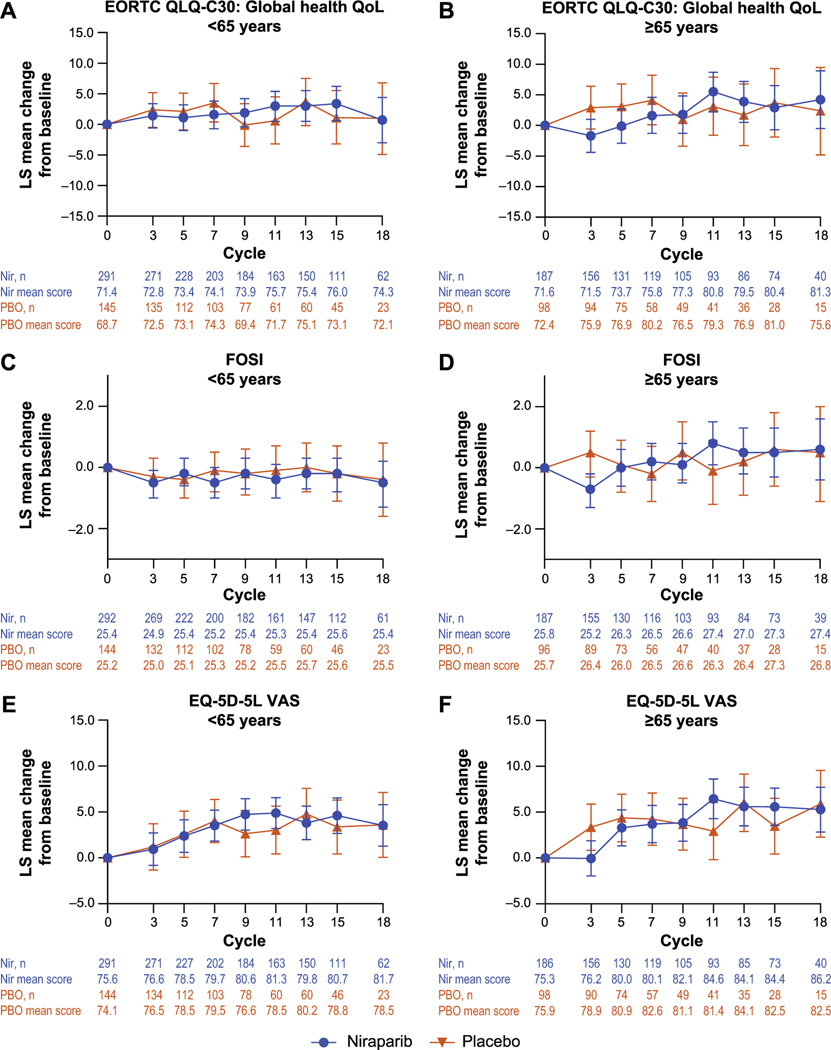
Patient-reported outcomes by age (<65 years vs ≥65 years). The LS mean change from baseline scores with 95% CI (represented by error bars) over time are reported for (A, B) the EORTC QLQ-C30 global health/overall QOL score, (C, D) FOSI, (E, F) EQ-5D-5L VAS, and (G, H) EORTC QLQ-OV28 abdominal/GI symptoms. The numbers underneath each graph detail the number of patients with data at each cycle and the mean score at each cycle for each treatment arm. Abbreviations: EORTC-QLQ-C30 = European Organisation for Research and Treatment of Cancer Quality of Life Questionnaire Core Questionnaire, EORTC-QLQ-OV28 = European Organisation for Research and Treatment of Cancer Quality of Life Questionnaire Ovarian Cancer Module, FOSI = Functional Assessment of Cancer Therapy Ovarian Symptom Index, GI = gastrointestinal, LS = least squares, Nir = niraparib, PBO = placebo, QOL = quality of life, VAS = visual analog scale.

Overall HRQOL and OC-specific symptoms were assessed by treatment arm for each age subgroup. LS mean change from baseline data are reported through cycle 18, which corresponds to the median follow-up time for the overall population (13.8 months). For patients aged ≥75 years, results are reported through cycle 15 because the small number of patients precluded analysis for cycle 18. Overall HRQOL assessed using the EORTC QLQ-C30 global health/overall QOL, FOSI, and EQ-5D-5L was similar between treatment arms in patients aged <65 years and in patients aged ≥65 years (Fig. 4). OC-specific symptoms were assessed using the EORTC QLQ-OV28 abdominal/gastrointestinal symptoms domain and showed similar results between treatment arms and across age subgroups (Fig. S4). Although the small number of patients aged ≥75 years makes interpretation difficult, PRO results for that group were generally similar to those for patients <75 years (Fig. S5). Because niraparib treatment is associated with anemia and gastrointestinal symptoms [35], EORTC QLQ-C30 PROs related to fatigue, a common symptom of anemia, and gastrointestinal symptoms were also evaluated by age at baseline (<65 years vs ≥65 years only). Through cycle 18, the LS mean change from baseline scores for EORTC QLQ-C30 physical function were similar across treatment arms and age subgroups; symptom scores for fatigue trended downward over time, indicating improvement, whereas pain scores were generally stable over time (Fig. S6). EORTC QLQ-C30 LS mean change from baseline scores for gastrointestinal symptoms (nausea and vomiting, appetite loss, and constipation) were higher (worse symptoms) in niraparib-treated patients than in placebo-treated patients in both age subgroups (Fig. S7). Except for constipation, differences between treatment arms resolved over time in both age subgroups. In the niraparib arm, constipation LS mean change from baseline scores trended downward from cycle 3 to cycle 7 but remained consistently higher than in the placebo arm through cycle 18; the separation between treatment arms was more pronounced in patients aged ≥65 years, with older niraparib-treated patients reporting higher scores (worse symptoms) than the younger patients. Diarrhea LS mean change from baseline scores trended higher (worse symptoms) in placebo-treated patients than in niraparib-treated patients over time in patients <65 years of age, although there was no separation between treatment arms (Fig. S7).

## 4. Discussion

In the PRIMA trial of niraparib first-line maintenance therapy in patients with newly diagnosed advanced OC, mPFS results were similar in patients aged <65 years and in patients aged ≥65 years and were consistent with the primary analysis results showing extended PFS duration with niraparib treatment compared with placebo [17]. A PFS benefit with niraparib treatment was observed in patients with HRd and HRp tumors in both age subgroups, with the greatest benefit compared with placebo observed in patients ≥65 years with HRd tumors. These findings were consistent with results from age-based post hoc analyses of the NOVA trial that evaluated niraparib maintenance therapy in patients with platinum-sensitive recurrent advanced OC [16]. In NOVA, there was an increased PFS benefit with niraparib treatment in patients aged ≥70 years compared with patients aged <70 years, particularly in patients in the germline *BRCA* mutant cohort [16].

The efficacy of niraparib in patients aged ≥65 years is also consistent with results for other PARP inhibitors in both the first-line and recurrent maintenance therapy settings. In an age-based post hoc analysis of the PAOLA-1/ENGOT-ov25 trial of first-line maintenance treatment with olaparib plus bevacizumab in patients with newly diagnosed advanced OC, the PFS benefit of olaparib was maintained in older patients [19]. Similar to PRIMA and NOVA, mPFS and hazard ratio data from PAOLA-1 also indicated an increased benefit of olaparib plus bevacizumab treatment in older patients with *BRCA* mutations or HRd disease [19]. The PFS benefit of PARP inhibitor maintenance therapy was also maintained in the overall population of older patients with platinum-sensitive recurrent advanced OC in the SOLO-2 trial of olaparib and the ARIEL3 trial of rucaparib [18,22].

In this analysis, the overall safety profile of niraparib was generally comparable between older and younger patients, with similar incidences of any-grade and grade ≥3 TEAEs reported across all 4 age subgroups examined. A numerically higher incidence of thrombocytopenia (any-grade and grade ≥ 3) was observed in older patients using both the 65-year and 75-year age cutoffs. Age-based variations in TEAEs were also reported in the placebo arm (ie, decreased incidence of insomnia and increased incidence of hypertension in patients aged ≥65 years compared with patients aged <65 years), indicating that underlying comorbidities or other patient population factors could have contributed to differences observed between the older and younger age subgroups. Consistent with overall population results [36], implementation of the niraparib ISD based on a patienťs baseline body weight and platelet count reduced the incidence of grade ≥3 events of thrombocytopenia, anemia, and neutropenia in patients in all age subgroups. In all cases, the reductions in TEAE incidence observed with the ISD were more pronounced in older patients, with the greatest effect observed in patients aged ≥75 years.

These results are consistent with the age-based post hoc analysis of NOVA that found that the niraparib safety profile was generally comparable between patients aged <70 years and ≥70 years at baseline [16]. Notably, in NOVA, older age was not associated with an increased incidence of thrombocytopenia in niraparib-treated patients [16]. Other PARP inhibitors have also reported similar safety profiles regardless of age. In PAOLA-1, the overall safety findings for olaparib plus bevacizumab were generally similar across age subgroups [19]. However, the incidence of grade ≥3 adverse events in the olaparib plus bevacizumab arm was slightly higher in patients aged ≥65 years than in patients <65 years, with older patients particularly experiencing a higher incidence of grade ≥3 events of hypertension and anemia [19]. In the recurrent setting, PARP inhibitor maintenance therapy safety was also similar across examined age subgroups. In SOLO-2, olaparib safety was generally similar in patients <65 years and in patients ≥65 years; however, the incidence of AML/MDS was notably higher in older patients [22]. A separate analysis from 8 complete prospective trials of olaparib showed trends toward increased hematologic toxicity with age [37]. In ARIEL3, the safety of rucaparib was generally similar across the 3 age groups examined, with a slightly higher incidence of grade ≥3 TEAEs reported in patients aged 65–74 years and ≥75 years than in patients aged <65 years [18].

Consistent with other PARP inhibitor trials [16,18,19,22], the incidence of patients discontinuing niraparib because of a TEAE was numerically higher in patients aged ≥65 years in our analysis. The trend toward higher discontinuation rates in older patients may reflect an age-related bias on behalf of investigators, as approximately 30% of niraparib patients aged ≥65 years who discontinued because of a TEAE did so because of events that were grade 1 or 2 in severity. In these patients, discontinuations could have occurred because the dose-reduction options allowed by the protocol were exhausted for TEAE management. Additionally, older patients could have been less willing to tolerate side effects overall, and persistent low-grade events of TEAEs such as nausea or fatigue could have negatively affected patient HRQOL and contributed to discontinuations. Similar results were observed in NOVA, where two-thirds of discontinuations due to TEAEs were for grade 1 or 2 events [16], as well as in an age-based analysis of first-line doublet chemotherapy from the AGO OVAR-3 study [38].

Because a patienťs treatment experience can impact their willingness to initiate and maintain treatment, HRQOL metrics are important to consider when evaluating treatment options. In this analysis, HRQOL metrics were similar across age subgroups, with older and younger patients generally reporting comparable experiences. Niraparib treatment did not adversely affect overall HRQOL, but it was associated with an early, largely transient worsening of gastrointestinal symptoms in both age subgroups. The only gastrointestinal symptom associated with niraparib treatment that did not resolve over time was constipation. Comparable HRQOL regardless of age is consistent with results from NOVA, SOLO-2, and ARIEL3, which found no marked difference in the treatment experience of older patients compared with younger patients [16,18,39]. Taken together, these results support the use of PARP inhibitor maintenance therapy in patients aged ≥65 years.

Understanding the impact of age on treatment outcomes remains complex. There is no universally accepted age cutoff to define older patients [40], and the varying age cutoffs used in studies make direct comparisons challenging. In addition, chronological age is an imperfect proxy that may not reflect a patienťs overall health status. Accordingly, measurements of frailty and other geriatric assessments that are more comprehensive and account for factors such as comorbidities, functional status, and social and emotional support can provide important insights into treatment outcomes and should also be considered to inform treatment decisions and optimize care [41–44].

This retrospective post hoc analysis was not prespecified or powered to determine differences between age subgroups, and its findings should be interpreted accordingly. The analysis did not account for known risk factors, such as *BRCA*m disease [7,8], that are associated with a younger age at diagnosis. Such analyses were beyond the scope of this article and would have been limited by small sample sizes for additional subgroups. The small number of patients in the ≥75-year age subgroup also limited the types of analyses that could be performed and the ability to meaningfully interpret the results for this population. Of note, older patients were underrepresented in the PRIMA population compared with the overall population of patients with OC. In PRIMA, approximately 40% of the study population was aged ≥65 years with only 10% aged ≥75 years. In contrast, approximately 47% and 23% of patients with OC are first diagnosed at ages ≥65 and ≥75 years, respectively [3]. The underrepresentation of older patients is consistent with other trials [16,18,38] and could be due to the highly selective nature of clinical trial enrollment, which often results in the exclusion of older patients because of comorbidities. Accordingly, these findings may not be generalizable to all older patients with OC. Moving forward, it will be important to design clinical trials to be more inclusive of older patients and patients who meet frailty or geriatric assessment criteria in order to provide additional insights into how PARP inhibitors work in these important patient populations.

## 5. Conclusions

In the PRIMA trial, niraparib treatment significantly prolonged PFS in patients with newly diagnosed advanced OC, and niraparib-treated patients experienced improved mPFS in all age subgroups evaluated. TEAE incidences were generally similar in patients aged <65 years and in patients aged ≥65 years, except for thrombocytopenia, which occurred more frequently in patients aged ≥65 years. The implementation of an ISD regimen reduced the incidence of hematologic TEAEs across age subgroups. PROs were similar across age subgroups, with an early, largely transient worsening of gastrointestinal symptoms observed in niraparib-treated patients. Collectively, these results demonstrate the efficacy and safety of niraparib first-line maintenance therapy in older patients enrolled in PRIMA and support the use of niraparib first-line maintenance therapy in patients with newly diagnosed advanced OC who respond to first-line PBC regardless of age.

## Supplementary Material

1

## Figures and Tables

**Table 1:** Baseline characteristics of the study population (<65 years and ≥65 years).

Characteristic, n (%)	Niraparib		Placebo	
		
	<65 years (*n* = 297)	≥65 years (*n* = 190)	<65 years (*n* = 147)	≥65 years (*n* = 99)

ECOG PS score				
0	218 (73.4)	119 (62.6)	108 (73.5)	66 (66.7)
1	79 (26.6)	71 (37.4)	39 (26.5)	33 (33.3)
FIGO stage				
III	192 (64.6)	126 (66.3)	89 (60.5)	69 (69.7)
IV	105 (35.4)	64 (33.7)	58 (39.5)	30 (30.3)
Best response to 1L PBC				
Complete response	206 (69.4)	131 (68.9)	102 (69.4)	70 (70.7)
Partial response	91 (30.6)	59 (31.1)	45 (30.6)	29 (29.3)
NACT				
Yes	187 (63.0)	135 (71.1)	93 (63.3)	74 (74.7)
No	110 (37.0)	55 (28.9)	54 (36.7)	25 (25.3)
Postoperative macroscopic residual disease status				
No visible residual disease	132 (44.4)	92 (48.4)	69 (46.9)	48 (48.5)
Visible residual disease	137 (46.1)	83 (43.7)	71 (48.3)	41 (41.4)
Missing/data not available	28 (9.4)	15 (7.9)	7 (4.8)	10 (10.1)
Tumor homologous recombination status				
HRd	173 (58.2)	74 (38.9)	88 (59.9)	38 (38.4)
HRp	88 (29.6)	81 (42.6)	41 (27.9)	39 (39.4)
HRnd	36 (12.1)	35 (18.4)	18 (12.2)	22 (22.2)
Tumor *BRCA* status				
*BRCA*m	109 (36.7)	43 (22.6)	54 (36.7)	17 (17.2)
*BRCA1*	88 (29.6)	17 (8.9)	36 (24.5)	7 (7.1)
*BRCA2*	21 (7.1)	26 (13.7)	18 (12.2)	10 (10.1)

Abbreviations: 1L = first-line, ECOG = Eastern Cooperative Oncology Group, FIGO = International Federation of Gynecology and Obstetrics, HRd = homologous recombination deficient, HRnd = homologous recombination not determined, HRp = homologous recombination proficient, NACT = neoadjuvant chemotherapy, PBC = platinum-based chemotherapy, PS = performance status.

**Table 2:** Summary of TEAEs and dose reductions, interruptions, and discontinuations by treatment arm and age (<65 years and ≥65 years).

	Niraparib		Placebo	
		
	<65 years (*n* = 294)	≥65 years (n = 190)	<65 years (*n* = 145)	≥65 years (n = 99)

Treatment exposure, months				
Median treatment exposure	11.2	9.7	8.3	8.3
Median duration of follow-up	15.1	14.6	14.8	14.2
TEAE summary, n (%)				
Any TEAE	289 (98.3)	189 (99.5)	136 (93.8)	88 (88.9)
Any grade ≥3 TEAE	203 (69.0)	138 (72.6)	28 (19.3)	18 (18.2)
Any serious TEAE	88 (29.9)	68 (35.8)	18 (12.4)	14 (14.1)
Any TEAE leading to death	2 (0.7)	0	0	1 (1.0)
Any TEAE leading to dose interruption	233 (79.3)	152 (80.0)	28 (19.3)	16 (16.2)
Any TEAE leading to treatment discontinuation	23 (7.8)	35 (18.4)	4 (2.8)	2 (2.0)

Abbreviations: TEAE = treatment-emergent adverse event.

## HIGHLIGHTS

- Age-based analysis of the efficacy and safety of niraparib first-line maintenance therapy in the phase 3 PRIMA trial.
- Niraparib first-line maintenance treatment improved median progression-free survival compared with placebo regardless of age.
- The incidences of any-grade and grade ≥3 treatment emergent adverse events were generally similar across all age subgroups.
- The individualized starting dose reduced grade ≥3 hematological treatment-emergent adverse events across all age subgroups
- Niraparib did not negatively impact health-related quality of life (HRQOL), and HRQOL was similar regardless of age.
