# Supplementary material for: Efficacy and safety of niraparib in patients aged 65 years and older with advanced ovarian cancer: Results from the PRIMA/ENGOT-OV26/GOG-3012 trial
Source: Gynecol Oncol. Author manuscript; Available in PMC 2025 May 30. (PMC12124154; doi:10.1016/j.ygyno.2024.03.009)
Supplement: 1 [file NIHMS2076701-supplement-1.pdf]

Supplemental Appendix

Supplementary materials have been provided by the authors to give readers additional information about their work.

Supplement to: Efficacy and Safety of Niraparib in Patients Aged 65 Years and Older with Advanced Ovarian Cancer: Results from the PRIMA/ENGOT-OV26/GOG-3012 Trial

Supplemental Appendix 1

Supplemental Tables and Figures... 2

Table S1. Baseline Characteristics of the Study Population by Treatment Arm and Age (<75 years and ≥75 years)... 2

Table S2. Independent Patient Characteristics Associated With PFS by Age in Niraparib-Treated Patients (<65 years and ≥65 years)... 3

Table S3. TEAE Resulting in Study Drug Withdrawal in Patients Aged ≥65 Years in the Niraparib Arm 4

Table S4. Summary of TEAEs and Dose Reductions, Interruptions, and Discontinuations by Treatment Arm and Age (<75 years and ≥75 years) 6

Figure S1. Efficacy (PFS) outcomes by age in patients <75 years and ≥75 years. 7

Figure S2. Most common TEAEs by treatment arm in patients aged <75 years and ≥75 years. 8

Figure S3. Selected grade ≥3 TEAEs in niraparib-treated patients by age (<75 years vs ≥75 years) and starting dose (FSD vs ISD)... 10

Figure S4. Patient-reported outcomes for ovarian cancer–specific symptoms by age (<65 years vs ≥65 years). 11

Figure S5. Patient-reported outcomes by age (<75 years vs ≥75 years) in the intent-to-treat population.. 12

Figure S6. EORTC QLQ-C30 domain scores for physical function, fatigue, and pain by age (<65 years vs ≥65 years) through cycle 18. 15

Figure S7. EORTC QLQ-C30 domain scores for gastrointestinal symptoms by age (<65 years vs ≥65 years) through cycle 18. 17

Supplemental Tables and Figures

Table S1. Baseline Characteristics of the Study Population by Treatment Arm and Age (<75 years and ≥75 years)

Characteristic, n (%)	Niraparib		Placebo	
	<75 years (n=433)	≥75 years (n=54)	<75 years (n=224)	≥75 years (n=22)
**ECOG PS score**				
0	309 (71.4)	28 (51.9)	162 (72.3)	12 (54.5)
1	124 (28.6)	26 (48.1)	62 (27.7)	10 (45.5)
**International FIGO stage**				
III	286 (66.1)	32 (59.3)	143 (63.8)	15 (68.2)
IV	147 (33.9)	22 (40.7)	81 (36.2)	7 (31.8)
**Best response to 1L PBC**				
Complete response	304 (70.2)	33 (61.1)	161 (71.9)	11 (50.0)
Partial response	129 (29.8)	21 (38.9)	63 (28.1)	11 (50.0)
**NACT**				
Yes	281 (64.9)	41 (75.9)	150 (67.0)	17 (77.3)
No	152 (35.1)	13 (24.1)	74 (33.0)	5 (22.7)
**Postoperative macroscopic residual disease status**				
No visible residual disease	199 (46.0)	25 (46.3)	109 (48.7)	8 (36.4)
Visible residual disease	196 (45.3)	24 (44.4)	101 (45.1)	11 (50.0)
Missing/data not available	38 (8.8)	5 (9.3)	14 (6.3)	3 (13.6)
**Tumor homologous recombination test status**				
HRd	222 (51.3)	25 (46.3)	120 (53.6)	6 (27.3)
HRp	148 (34.2)	21 (38.9)	71 (31.7)	9 (40.9)
HRnd	63 (14.5)	8 (14.8)	33 (14.7)	7 (31.8)
**Tumor *BRCA* status**				
*BRCA*m	143 (33.0)	9 (16.7)	67 (29.9)	4 (18.2)
BRCA1	100 (23.1)	5 (9.3)	40 (17.9)	3 (13.6)
BRCA2	43 (9.9)	4 (7.4)	27 (12.1)	1 (4.5)

Abbreviations: 1L = first-line, ECOG = Eastern Cooperative Oncology Group, FIGO = International Federation of Gynaecology and Obstetrics, HRd = homologous recombination deficient, HRnd = homologous recombination not determined, HRp = homologous recombination proficient, NACT = neoadjuvant chemotherapy, PBC = platinum-based chemotherapy, PS = performance status.

Table S2. Independent Patient Characteristics Associated With PFS by Age in Niraparib-Treated Patients (<65 years and ≥65 years)

Age	Independent factor[a]	Hazard ratio for PFS (95% CI)	*P* value
<65 years	HRd compared with HRp/HRnd	0.31 (0.21–0.43)	<0.0001
≥65 years	Best response to 1L treatment of CR compared with PR	0.50 (0.33–0.76)	0.0010
	HRd compared with HRp/HRnd	0.35 (0.22–0.56)	<0.0001

[a]Identified via backward selection procedure using a 0.05 significance level.

Abbreviations: 1L = first-line, CR = complete response, HRd = homologous recombination deficient, HRp = homologous recombination proficient, HRnd = homologous recombination not determined, PFS = progression-free survival, PR = partial response.

Table S3. TEAE Resulting in Study Drug Withdrawal in Patients Aged ≥65 Years in the Niraparib Arm

Patient age, years	Weight, kg	ECOG PS score	Symptom/toxicity (MedDRA preferred term)	Severity grade	HRD status
74	57.4	1	Gamma-glutamyltransferase increased	3	HRd
			Neutropenia	3	HRd
71	62.3	0	Asthenia	2	HRd
			Respiratory tract infection	2	
			Thrombocytopenia	4	
			Thrombocytopenia	1	
			Viral upper respiratory tract infection	1	
75	45.5	1	Nausea	2	HRd
66	52.5	1	Nausea	1	HRd
72	52	0	Thrombocytopenia	4	HRd
71	65	0	Decreased appetite	1	HRd
			Dizziness	1	
			Fatigue	1	
69	115.2	1	Flushing	1	HRd
			Nausea	1	
67	89	1	Nausea	3	HRd
74	75.5	0	Thrombocytopenia	4	HRd
76	90.3	1	Thrombocytopenia	4	HRd
82	57.2	1	Invasive ductal breast carcinoma	3	HRd
74	65.6	0	Thrombocytopenia	3	HRd
			Thrombocytopenia	4	
65	80.4	0	Anemia	2	HRd
70	64.2	1	Pneumonia	3	HRp
75	53	0	Decreased appetite	1	HRp
			Diarrhea	1	
			Dizziness	1	
			Headache	1	
			Nausea	1	
68	66	0	Neutrophil count decreased	3	HRp
66	102	1	Abdominal pain	3	HRp
			Nausea	1	
			Thrombocytopenia	1	
			Thrombocytopenia	3	
			Weight decreased	1	

75	83	1	Blood creatinine increased	3	HRp
69	68	1	Anemia<br>Neutropenia<br>Neutropenia	2<br>3<br>3	HRp
66	55	1	Neutropenia	4	HRp
73	55	0	Thrombocytopenia	4	HRp
74	80	0	General physical health deterioration	3	HRp
68	76.9	0	Cellulitis	3	HRp
68	80.4	1	Peripheral sensory neuropathy	2	HRp
72	74.4	1	Anemia<br>Neutropenia<br>Thrombocytopenia	2<br>3<br>3	HRp
79	79.3	0	Anemia	3	HRp
65	86.4	0	Thrombocytopenia	4	HRp
79	52.5	1	Anemia	3	HRp
68	89.8	0	Depression<br>Fatigue	2<br>3	HRp
71	49	0	Asthenia	3	HRnd
70	82	0	Neutropenia	2	HRnd
73	103	0	Anemia	3	HRnd
80	59	0	Anxiety<br>Hallucination<br>Insomnia	3<br>3<br>3	HRnd
77	61.1	1	Thrombocytopenia	2	HRnd
78	77.8	1	Neutrophil count decreased	2	HRnd

Abbreviations: ECOG = Eastern Cooperative Oncology Group, HRd = homologous recombination deficient, HRp = homologous recombination proficient, HRnd = homologous recombination not determined, MedDRA = Medical Dictionary for Regulatory Activities, PS = performance score.

Table S4. Summary of TEAEs and Dose Reductions, Interruptions, and Discontinuations by Treatment Arm and Age (<75 years and ≥75 years)

	Niraparib		Placebo	
	<75 years (n=430)	≥75 years (n=54)	<75 years (n=222)	≥75 years (n=22)
**Treatment exposure, months**				
Median treatment exposure	11.1	11.0	8.3	4.9
Median duration of follow-up	15.0	14.0	14.8	13.6
**TEAE summary, n (%)**				
Any TEAE	424 (98.6)	54 (100.0)	202 (91.0)	22 (100.0)
Any grade ≥3 TEAE	303 (70.5)	38 (70.4)	42 (18.9)	4 (18.2)
Any serious TEAE	136 (31.6)	20 (37.0)	29 (13.1)	3 (13.6)
Any TEAE leading to death	2 (0.5)	0	0	1 (4.5)
Any TEAE leading to dose interruption	340 (79.1)	45 (83.3)	41 (18.5)	3 (13.6)
Any TEAE leading to treatment discontinuation	48 (11.2)	10 (18.5)[a]	5 (2.3)	1 (4.5)

[a]Four patients discontinued because of a grade 1 or 2 TEAE.

Abbreviations: TEAE = treatment-emergent adverse event.

Figure S1. Efficacy (PFS) outcomes by age in patients <75 years and ≥75 years.

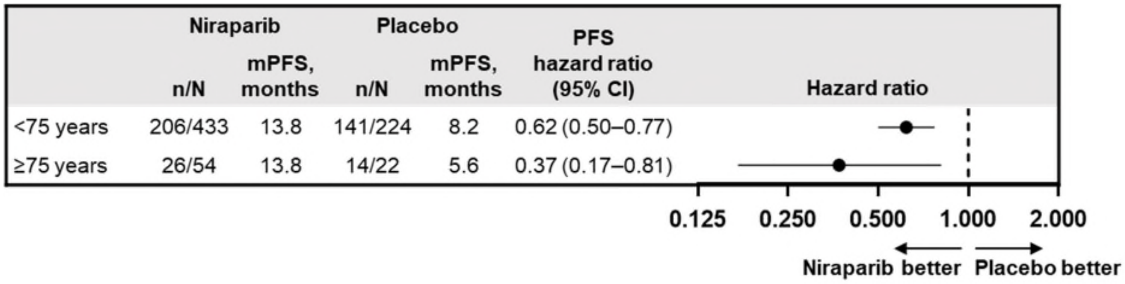

	Niraparib		Placebo		PFS hazard ratio (95% CI)
	n/N	mPFS, months	n/N	mPFS, months	
<75 years	206/433	13.8	141/224	8.2	0.62 (0.50–0.77)
≥75 years	26/54	13.8	14/22	5.6	0.37 (0.17–0.81)

Figure S1. Efficacy (PFS) outcomes by age in patients <75 years and ≥75 years. Assessment by tumor HRD status was not performed because of the small sample size for patients aged ≥75 years.

Abbreviations: HRD = homologous recombination deficiency, m = median, PFS = progression-free survival.

Figure S2. Most common TEAEs by treatment arm in patients aged <75 years and ≥75 years.

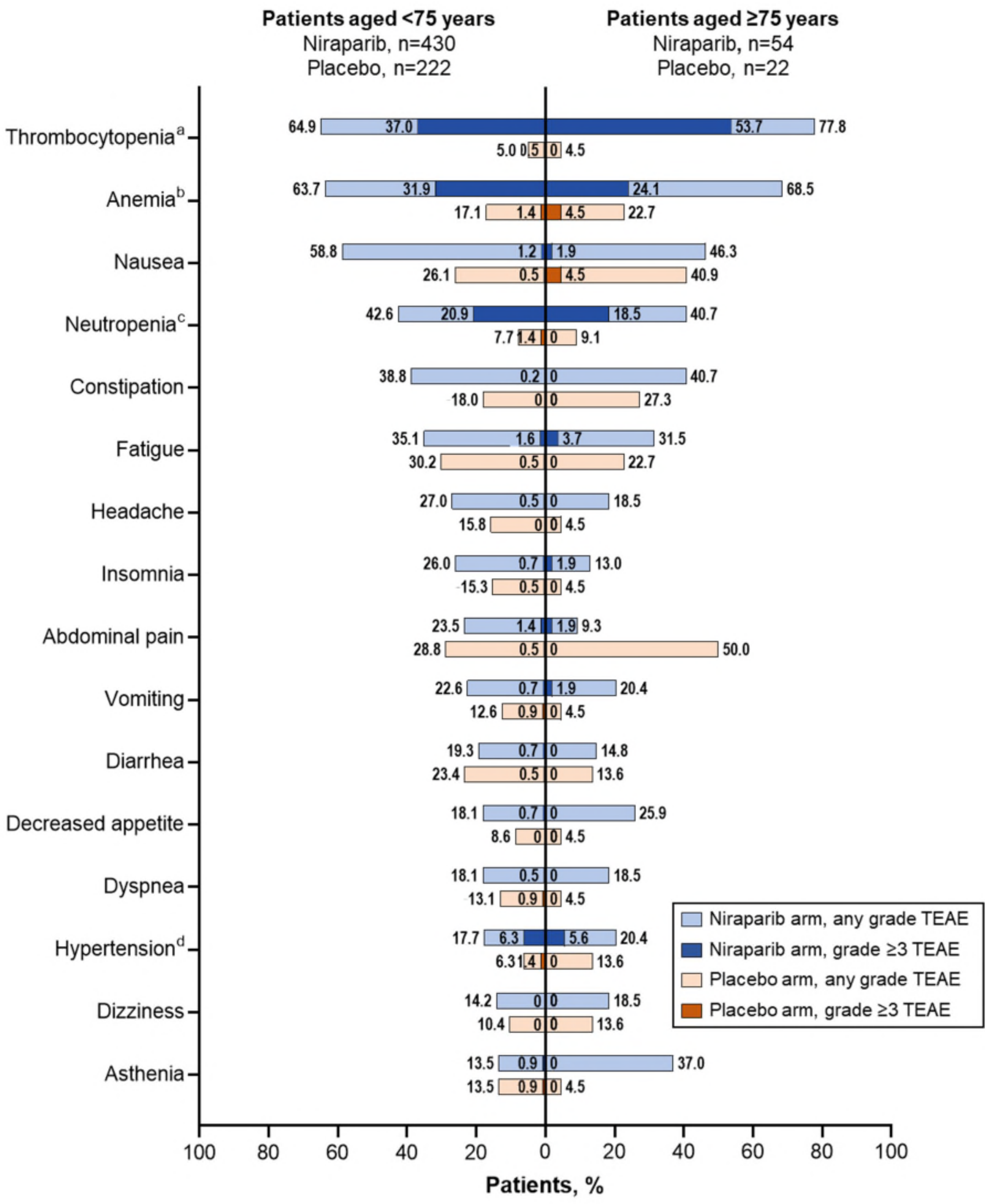

Figure S2. Most common TEAEs by treatment arm in patients aged <75 years and ≥75 years. TEAEs reported in ≥20% of niraparib-treated patients for any age subgroup (<65 years, ≥65 years, <75 years, ≥75 years).

[a]Includes thrombocytopenia and platelet count decreased.

[b]Includes anemia, hematocrit decreased, hemoglobin decreased, red blood cell decreased, and macrocytic anemia.

[c]Includes neutropenia, neutrophil count decreased, neutropenic sepsis, and febrile neutropenia.

[d]Includes hypertension and blood pressure increased.

Abbreviations: TEAE = treatment-emergent adverse event.

Figure S3. Selected grade ≥3 TEAEs in niraparib-treated patients by age (<75 years vs ≥75 years) and starting dose (FSD vs ISD).

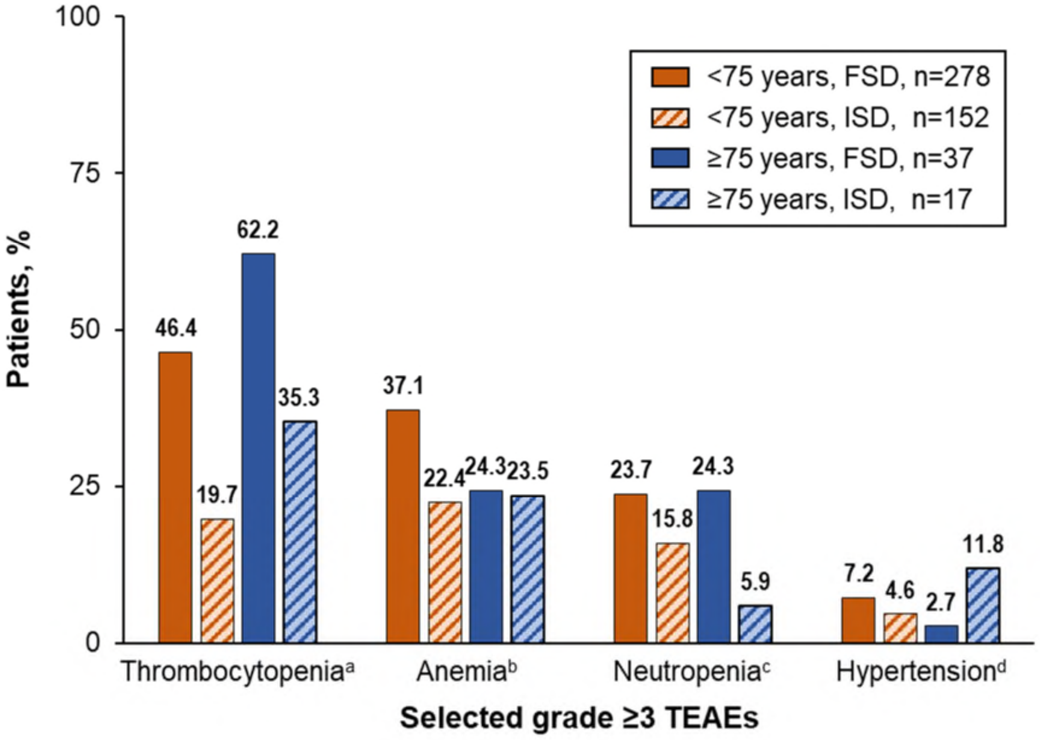

Figure S3. Selected grade ≥3 TEAEs in niraparib-treated patients by age (<75 years vs ≥75 years) and starting dose (FSD vs ISD).

[a]Includes thrombocytopenia and platelet count decreased.

[b]Includes anemia, hematocrit decreased, hemoglobin decreased, red blood cell decreased, and macrocytic anemia.

[c]Includes neutropenia, neutrophil count decreased, neutropenic sepsis, and febrile neutropenia.

[d]Includes hypertension and blood pressure increased.

Abbreviations: FSD = fixed starting dose, ISD = individualized starting dose, TEAE = treatment-emergent adverse event.

Figure S4. Patient-reported outcomes for ovarian cancer–specific symptoms by age (<65 years vs ≥65 years).

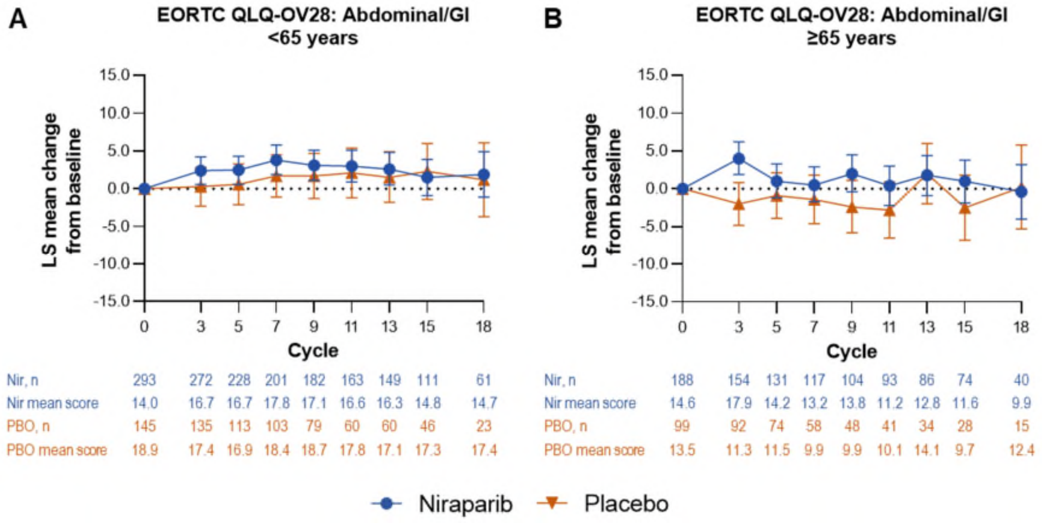

Figure S4. Patient-reported outcomes for ovarian cancer–specific symptoms by age (<65 years vs ≥65 years). The LS mean change from baseline scores with 95% CI (represented by error bars) over time are reported for (A, B) the EORTC QLQ-OV28 abdominal/GI symptoms. The numbers underneath each graph detail the number of patients with data at each cycle and the mean score at each cycle for each treatment arm.

Abbreviations: EORTC-QLQ-OV28 = European Organisation for Research and Treatment of Cancer Quality of Life Questionnaire Ovarian Cancer Module, GI = gastrointestinal, LS = least squares, Nir = niraparib, PBO = placebo.

Figure S5. Patient-reported outcomes by age (<75 years vs ≥75 years) in the intent-to-treat population.

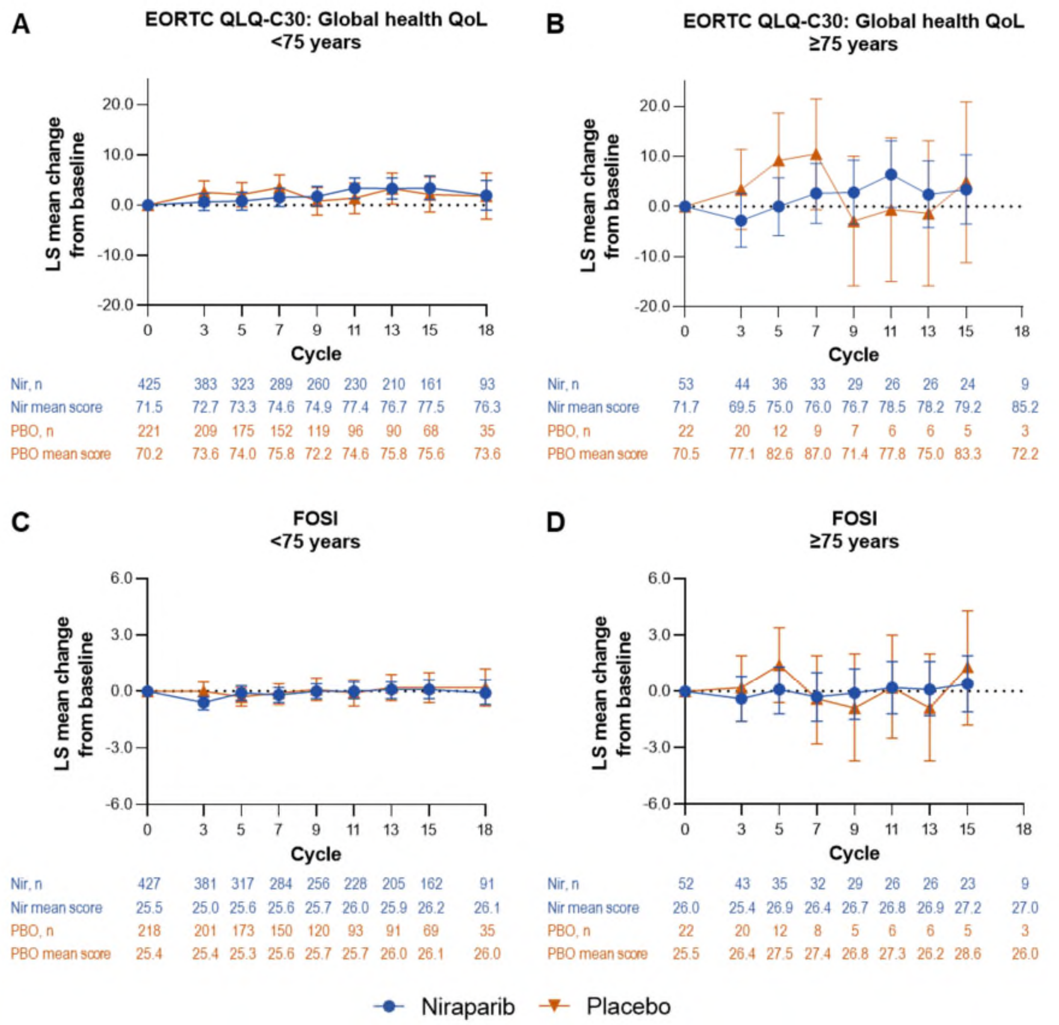

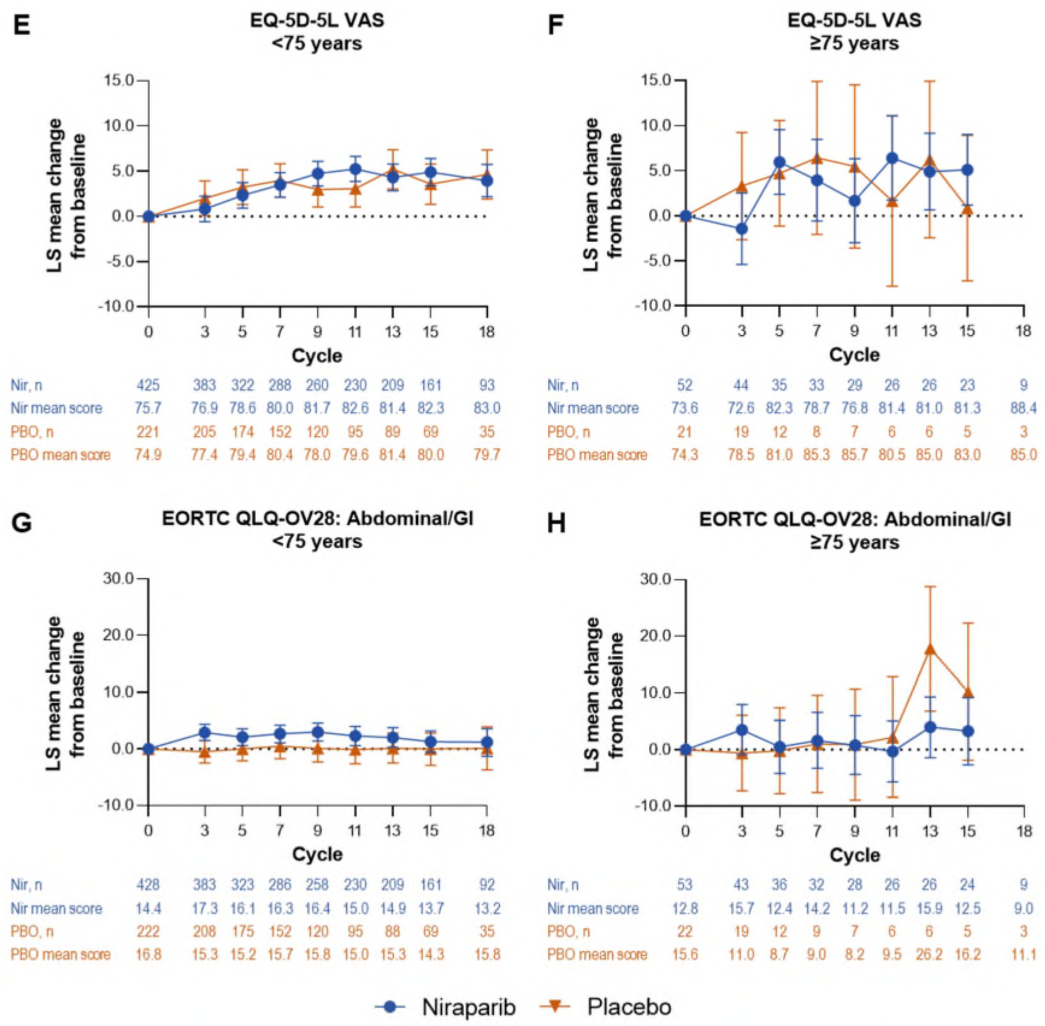

Figure S5. Patient-reported outcomes by age (<75 years vs ≥75 years) in the intent-to-treat population. The LS mean change from baseline scores with 95% CI (represented by error bars) over time are reported for (A, B) the EORTC QLQ-C30 global health/overall QOL score, (C, D) FOSI, (E, F) EQ-5D-5L VAS, and (G, H) EORTC QLQ-OV28 abdominal/GI symptoms. The numbers underneath each graph detail the number of patients with data at each cycle and the mean score at each cycle for each treatment arm.

Abbreviations: EORTC-QLQ-C30 = European Organisation for Research and Treatment of Cancer Quality of Life Questionnaire Core Questionnaire, EORTC-QLQ-OV28 = European Organisation for Research and Treatment of Cancer Quality of Life Questionnaire Ovarian Cancer Module, FOSI = Functional Assessment of Cancer Therapy Ovarian Symptom Index,

GI = gastrointestinal, LS = least squares, Nir = niraparib, PBO = placebo, QOL = quality of life, VAS = visual analog scale.

Figure S6. EORTC QLQ-C30 domain scores for physical function, fatigue, and pain by age (<65 years vs ≥65 years) through cycle 18.

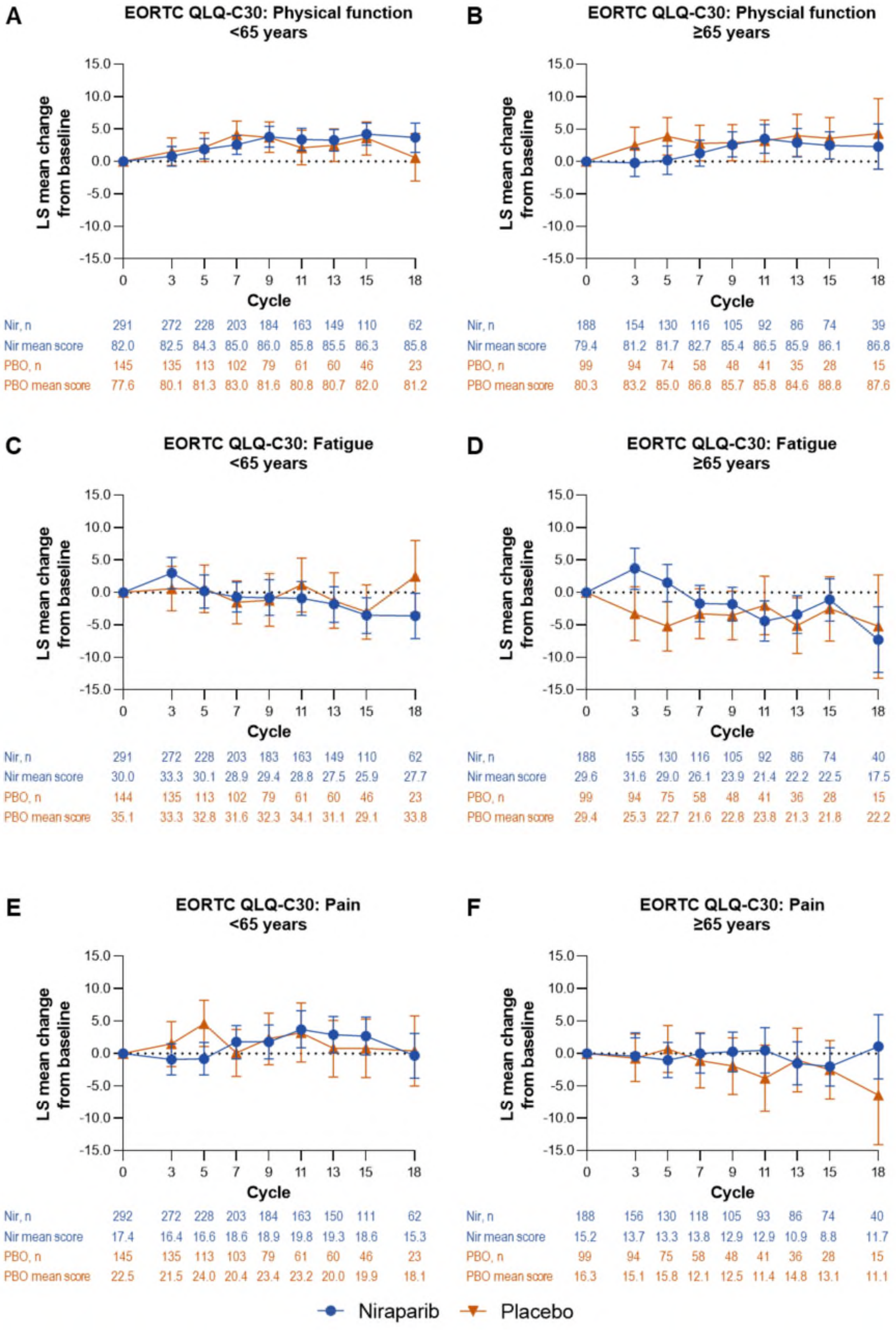

Figure S6. EORTC QLQ-C30 domain scores for physical function, fatigue, and pain by age (<65 years vs ≥65 years) through cycle 18. The LS mean change from baseline scores with 95% CI (represented by error bars) over time are reported for (A, B) physical function and symptom-specific scores for (C, D) fatigue and (E, F) pain. The numbers underneath each graph detail the number of patients with data at each cycle and the mean score at each cycle for each treatment arm.

Abbreviations: EORTC QLQ-C30 = European Organisation for Research and Treatment of Cancer Quality of Life Questionnaire Core Questionnaire, LS = least squares, QOL = quality of life.

Figure S7. EORTC QLQ-C30 domain scores for gastrointestinal symptoms by age (<65 years vs ≥65 years) through cycle 18.

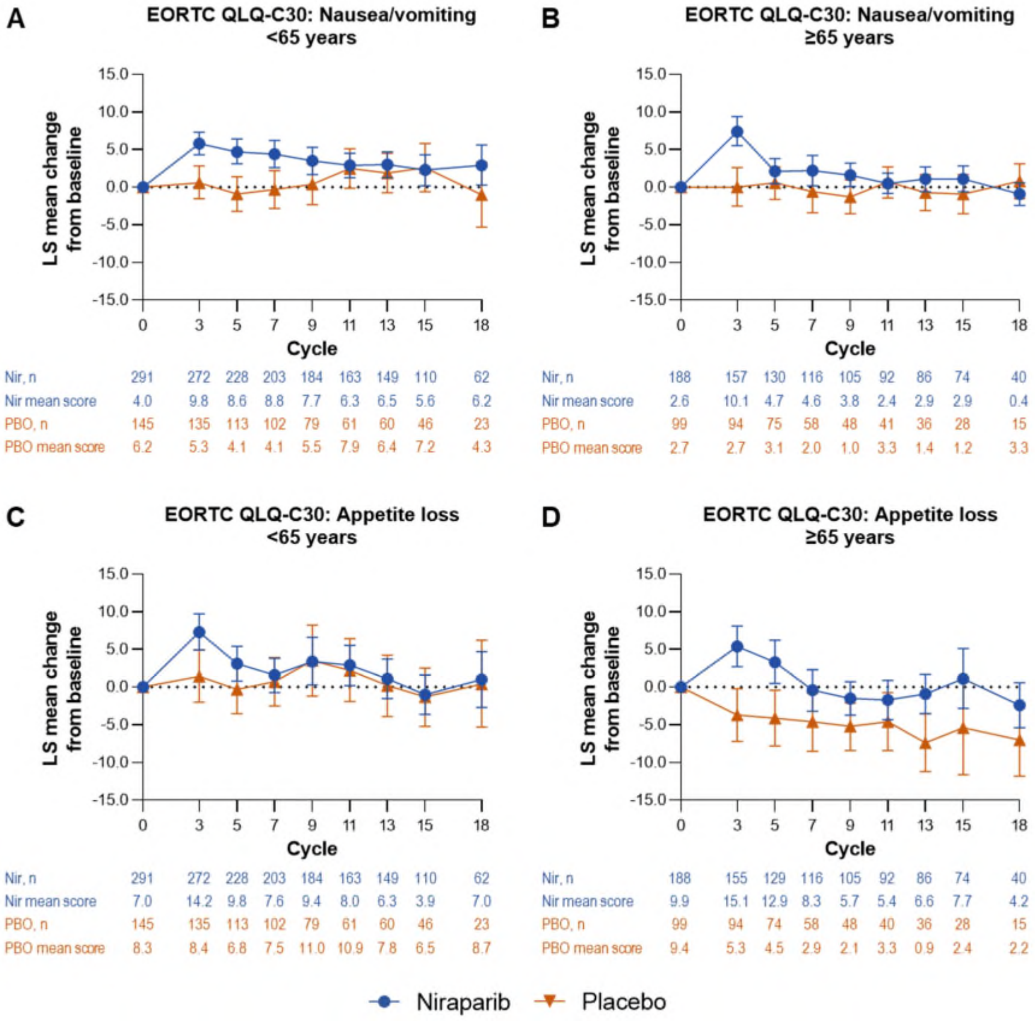

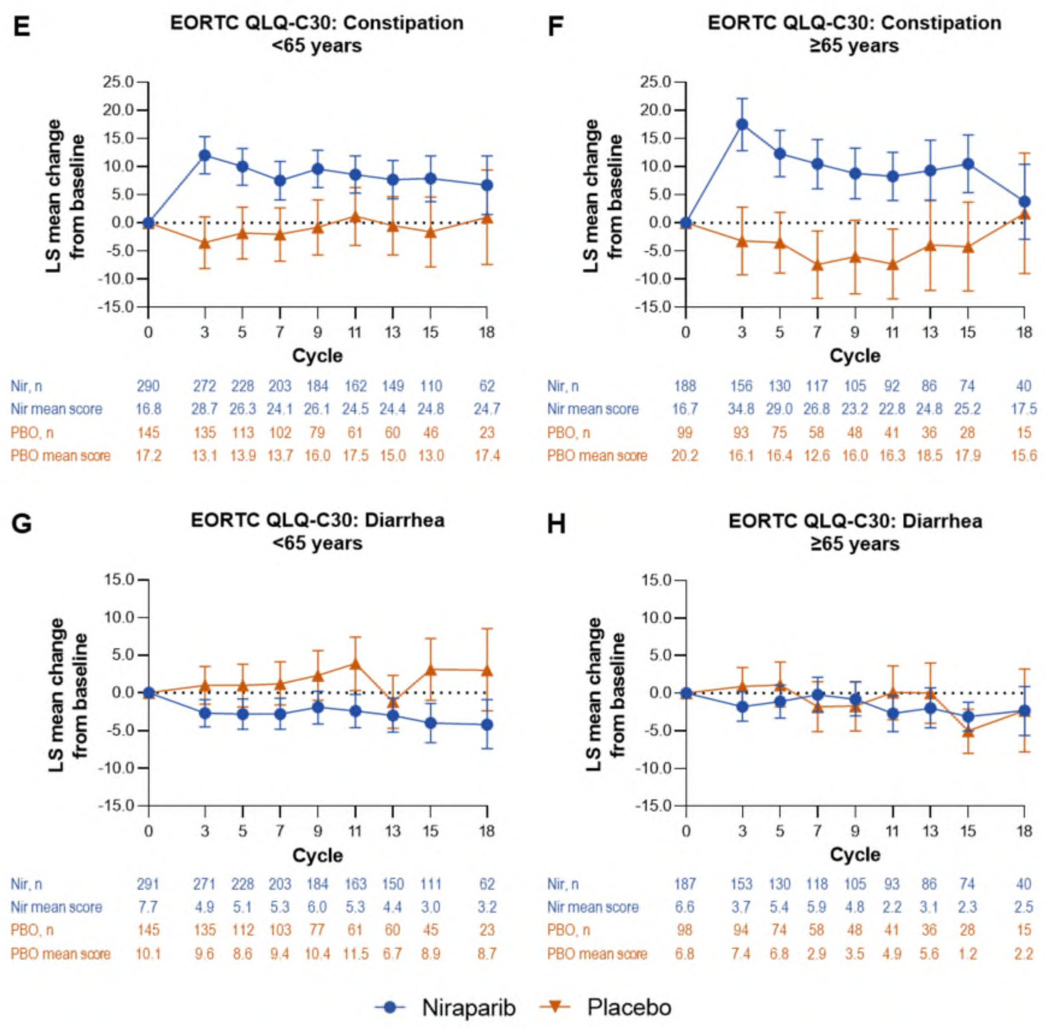

Figure S7. EORTC QLQ-C30 domain scores for gastrointestinal symptoms by age (<65 years vs ≥65 years) through cycle 18. The LS mean change from baseline scores with 95% CI (represented by error bars) over time are reported for (A, B) nausea and vomiting, (C, D) appetite loss, (E, F) constipation, and (G, H) diarrhea. The numbers underneath each graph detail the number of patients with data at each cycle and the mean score at each cycle for each treatment arm.

Abbreviations: EORTC QLQ-C30 = European Organisation for Research and Treatment of Cancer Quality of Life Questionnaire Core Questionnaire, LS = least squares, Nir = niraparib, PBO = placebo.
